# High-Resolution Imaging of Polyethylene Glycol Coated Dendrimers via Combined Atomic Force and Scanning Tunneling Microscopy

**DOI:** 10.1155/2015/535683

**Published:** 2015-01-01

**Authors:** Shawn Riechers, Qian Zhong, Nai-Ning Yin, Arpad Karsai, Sandro R. P. da Rocha, Gang-yu Liu

**Affiliations:** ^1^Department of Chemistry, University of California, Davis, CA 95616, USA; ^2^Department of Chemical Engineering & Materials Science, Wayne State University, Detroit, MI 48202, USA

## Abstract

Dendrimers have shown great promise as drug delivery vehicles in recent years because they can be synthesized with designed size and functionalities for optimal transportation, targeting, and biocompatibility. One of the most well-known termini used for biocompatibility is polyethylene glycol (PEG), whose performance is affected by its actual conformation. However, the conformation of individual PEG bound to soft materials such as dendrimers has not been directly observed. Using atomic force microscopy (AFM) and scanning tunneling microscopy (STM), this work characterizes the structure adopted by PEGylated dendrimers with the highest resolution reported to date. AFM imaging enables visualization of the individual dendrimers, as well as the differentiation and characterization of the dendrimer core and PEG shell. STM provides direct imaging of the PEG extensions with high-resolution. Collectively, this investigation provides important insight into the structure of coated dendrimers, which is crucial for the design and development of better drug delivery vehicles.

## 1. Introduction 

Dendrimers provide an alternative and potent means for drug delivery due to their nanometer size and the ability to incorporate various functionalities on their interior and exterior. Modern chemical synthesis capabilities allow various functionalities to be incorporated on the dendrimer exterior in order to optimize performance in terms of drug binding, transport, targeting, delivery, and biocompatibility [1–3]. Polyamidoamine (PAMAM) dendrimers, for example, have been tailored for enhanced drug solubility, retention time, targeting, and efficacy [1–3]. In addition to optimizing delivery, another issue is the reduction or elimination of cytotoxicity, which has been addressed by masking the terminal functional groups and charge [4]. This can be accomplished by adding biologically compatible terminal groups such as carboxylate, hydroxyl, acetamide, lipid, or polyethylene glycol (PEG) [2]. Among these, PEG is the most widely used due to its minimal or nontoxicity, nonimmunogenicity, and nonantigenicity and has been approved by the FDA in oral intravenous and pulmonary pharmaceutical formulations [5–8]. It is known that PEG chains adopt a variety of conformations, lengths, and packing density and that these structural presentations directly affect biocompatibility [9–11]. In some cases, PEG alters dendrimer's drug loading capacity, retention time, and thus their delivery performance [8, 12, 13]. Therefore, the characterization of PEG coating prior to animal testing is of great importance. While computational studies have been carried out to probe PEG conformation on dendrimers surfaces, experimental studies are lacking due to the difficulties in obtaining high-resolution structural characterization of PEG when bound to soft materials, such as dendrimers [11, 14]. Our prior work shows that high-resolution structural characterization of simple dendrimers such as PAMAM can be achieved by advanced sample preparation and combined atomic force and scanning tunneling microscopy (AFM and STM) imaging [15, 16]. Encouraged by this initial success, this work reports extending our approach to PEGylated PAMAM dendrimers. From the high-resolution images, the molecular conformation, packing density, and distribution of PEG on the surface of individual dendrimers can be obtained. This knowledge of the PEG presentation on dendrimers provides important insights for understanding structure-delivery performance correlation, which could guide the design, optimization, and development of the next generation of dendrimers and reduce usage of animal tests.

## 2. Materials and Methods

### 2.1. Materials

The following materials were used as received: methoxy polyethylene glycol 1000 Da (PEG_1000_) (Sigma-Aldrich), triethylamine (TEA) (99.5%, Sigma-Aldrich), pyridine (99.8%, Sigma-Aldrich) p-nitrophenyl chloroformate (pNPCF) (98%, Sigma-Aldrich), 2,5-dihydroxybenzoic acid (2,5-DHB) (Sigma-Aldrich), anhydrous dichloromethane (DCM) (Sigma-Aldrich), dimethyl sulfoxide (DMSO) (Acros Geel), K_2_PtCl_4_ (min. 42.4% Pt, Alfa Aesar), n-octanethiol (C8) (98%, Sigma-Aldrich), deuterium oxide (D, 99.96%, Cambridge Isotope Laboratories), and phosphate buffer (10x, Lief Technologies). Fourth generation amine terminated PAMAM dendrimers were purchased as 10% by weight solutions in methanol (Sigma-Aldrich, St. Louis, MO). Silica gel 60 A 230–400 mesh ATSM (Whatman Inc) and silica gel 60 F_254_ plastic sheets (TLC) (Merck KGaA) were used for column and thin layer chromatography, respectively. Ultrapure water (≥18 MΩ·cm, Millipore Milli-Q) and 200 proof ethanol (Gold Shield Chemical Co.) were used for dilution and washing. Ultrapure N_2_ (98%, Air Gas Co.) and H_2_ (99.99%, Praxair, Inc.) were used for drying and flaming, respectively. Tungsten wire (*d* = 0.010 in., 99.95%, California Fine Wire Co.) was used to make STM tips. Au slugs (99.99%, Alpha Aesar Premion Co.) and mica (clear ruby muscovite, Mica New York Corp.) were used for Au thin film preparation.

### 2.2. Methods

#### 2.2.1. Preparation of Gold Thin Films

The following materials were used as received: methoxy polyethylene glycol 1000 Da (PEG1000) (Sigma-Aldrich, St. Louis, MO), triethylamine (TEA) (99.5%, Sigma-Aldrich, St. Louis, MO), pyridine (99.8%, Sigma-Aldrich, St. Louis, MO) p-nitrophenyl chloroformate (pNPCF) (98%, Sigma-Aldrich, St. Louis, MO), 2,5-dihydroxybenzoic acid (2,5-DHB) (Sigma-Aldrich, St. Louis, MO), anhydrous dichloromethane (DCM) (Sigma-Aldrich, St. Louis, MO), dimethyl sulfoxide (DMSO) (99.7%, Acros, Geel, Belgium), K2PtCl4 (min. 42.4% Pt, Alfa Aesar, Ward Hill, Massachusetts), n-octanethiol (C8) (98%, Sigma-Aldrich, St. Louis, MO), deuterium oxide (D, 99.96%, Cambridge Isotope Laboratories, Tewksbury, MA), and phosphate buffer (10x, Lief Technologies, Grand Island, NY). Fourth generation amine terminated PAMAM dendrimers were purchased as 10% by weight solutions in methanol (Sigma-Aldrich, St. Louis, MO). Silica gel 60A 230–400 mesh ATSM (Whatman Inc, Pittsburgh, PA) and silica gel 60 F254 plastic sheets (TLC) (Merck KGaA, Darmstadt, Germany) were used for column and thin layer chromatography, respectively. Ultrapure water (≥18MΩ·cm, MilliporeMilli-Q, Billerica, MA) and 200 proof ethanol (Gold Shield Chemical Co., Hayward, CA) were used for dilution and washing. Ultrapure N2 (98%, Air Gas Co., Woodland, CA) and H2 (99.99%, Praxair Inc., Sacramento, CA) were used for drying and flaming, respectively. Tungsten wire (*d* = 0.010 in., 99.95%, California Fine Wire Co., Grover Beach, CA) was used to make STM tips. Au slugs (99.99%, Alpha Aesar Premion Co., Ward Hill MA) and mica (clear ruby muscovite, Mica New York Corp., New York, NY) were used for Au thin film preparation.

#### 2.2.2. PEGylation of G4-PAMAM-NH_2_ Dendrimers

G4-PAMAM-NH_2_ dendrimers were PEGylated according to previous reports with some modifications [13]. Briefly, PEG_1000_ was first modified by reaction with TEA and a catalyst amount of pyridine in anhydrous DCM with PEG_1000_. To the organic solution was added pNPCF dropwise. The organic solvent was then removed under reduced pressure. To remove unreacted pNPCF, the resultant product was purified by column chromatography.

The G4-PAMAM-*n*PEG_1000_ was synthesized by adding an anhydrous DMSO solution of G4-PAMAM-NH_2_ dropwise to a solution of PEG_1000_ carbonate in anhydrous DMSO. The reaction system was then stirred 24 hours for G4-PAMAM-6PEG_1000_, or 72 hours for G4-PAMAM-50PEG_1000_. The resulting G4-PAMAM-*n*PEG_1000_ was purified by centrifugal filter (MWCO = 10 kDa) until thin layer chromatography (DCM/methanol = 80/20, v/v) showed no unreacted PEG_1000_ carbonate or byproduct p-nitrophenol.

#### 2.2.3. G4-PAMAM-*n*PEG_1000_ Characterization

The molecular weight of the modified and unmodified dendrimers was determined by matrix assisted laser desorption ionization time-of-flight mass spectrometry (MALDI-TOF-MS) (Ultraflex, Bruker). Spectra were acquired under positive ion reflector mode. The conjugates were dissolved in deionized water at a concentration of 1.0 mg/mL. 10 mg/mL of 2,5-DHB in methanol was used as matrix. 10 *μ*L of the conjugate solution was mixed with 10 *μ*L of the DHB solution. 2 *μ*L of the sample was spotted on a MALDI target plate (MTP 384, Bruker Daltonics, Inc.). The spotted sample was dried gently by air flow. The G4-PAMAM-NH_2_, G4-PAMAM-6PEG_1000_, and G4-PAMAM-50PEG_1000_ dendrimers were found to have a molecular weight of 14,579, 22,080, and 75,929 Da, respectively.

The number of PEG chains per dendrimer was determined by ^1^H NMR spectroscopy (MR-400, Agilent) using deuterated solvent. The deuterated solvent peak (DMSO_d6: 2.483; D_2_O: 4.577) in ^1^H NMR was set as a reference peak. The PEGylation resulted in 5.9 PEG per dendrimer, referred to as G4-PAMAM-6PEG_1000_, and 50.5 PEG per dendrimer, referred to as G4-PAMAM-50PEG_1000_.

The hydrodynamic diameter (HD) of samples was measured using dynamic light scattering (DLS) (Zetasizer Nano ZS, Melvern Instruments). The sample (1.0 mg/mL) was dissolved in phosphate buffer solution (0.1 M, pH 7.4) to maintain the pH during the measurement. HD and standard deviations were automatically calculated by built-in software. G4-PAMAM-NH_2_, G4-PAMAM-6PEG_1000_, and G4-PAMAM-50PEG_1000_ dendrimers were found to have a HD of 4.4 ± 1.4, 5.8 ± 2.1, and 12.2 ± 4.4 nm, respectively.

#### 2.2.4. AFM and STM Imaging

Dendrimers were immobilized on gold surfaces for AFM and STM imaging. The solutions were prepared following previously established procedures, including metal ion doping to facilitate STM imaging [15–17]. In short, dendrimer solutions were diluted to 1 *μ*M in pure water. K_2_PtCl_4_ was then added to obtain molar ratios of 1 : 70, or 1 : 700 dendrimer : Pt^2+^. Solutions were kept at room temperature for 48 hours in order to allow for sufficient Pt^2+^-amine coordination within dendrimers. For the surface deposition of dendrimers, 1 cm^2^ pieces of gold films were H_2_-flamed and allowed to cool for 10 minutes under clean ambient conditions. Then, a 60–100 *μ*L drop of the dendrimer solution was deposited resulting in a droplet with a contact diameter of 0.7–0.9 cm. After 1.25 minutes the surface was washed with 2 mL of pure water, dried with N_2_, washed with 2 mL of ethanol, and dried again with N_2_. A 60–100 *μ*L drop of 1.0 mM C8 solution was then applied to the substrate for 4 minutes, washed with 2 mL of ethanol, and dried with N_2_. C8 SAMs are incorporated in order to replace weakly adsorbed molecules, confine dendrimers laterally, and prevent mobility during scanning. This process results in a clean, high coverage submonolayer of dendrimers on the surface.

AFM images were acquired using a MFP3D-SA system (Asylum Research), which includes a closed loop capability. A silicon cantilever (AC-240, Olympus) was used for imaging and nanoshaving. The probe has a typical force constant of *k* = 1.0 N/m as measured by the thermal noise method [16, 18, 20, 21]. During tapping mode imaging, the cantilever was modulated by a driving frequency of 74 kHz and amplitude of 87 nm (1.0 V). During nanoshaving to displace adsorbates such as dendrimers or alkanethiolates, tips were placed in contact with the surface with increasing load beyond threshold [22, 23]. Image processing and data analysis were performed using Gwyddion (Version 2.33, http://gwyddion.net/).

STM images were taken using a walker-type scanner (UHV 300, RHK Technologies, Inc.), under ambient pressure and temperature [18, 19]. STM tips were prepared by etching tungsten wires electrochemically at 2.0 V in 3.0 M NaOH solutions using a homemade potentiostat to monitor the etching process [18, 19]. All STM images were acquired in constant current mode with typical bias voltages ranging from 0.4 to 0.9 V and tunneling currents from 10 to 40 pA. The scanner was calibrated laterally using an octanethiol SAM lattice constant of 0.50 nm and vertically using Au(111) single atomic step (0.235 nm). Image processing and data analysis were performed using XPMPro (Version 2.0.0.8, RHK Technologies, Inc.). Surface contact area measurements were carried out using ImageJ (Version 1.47v, Wayne Rasband, NIH, http://imagej.nih.gov/ij/index.html).

## 3. Results and Discussion

### 3.1. AFM Imaging of the PEGylated G4 Dendrimers

**Figure 1:**
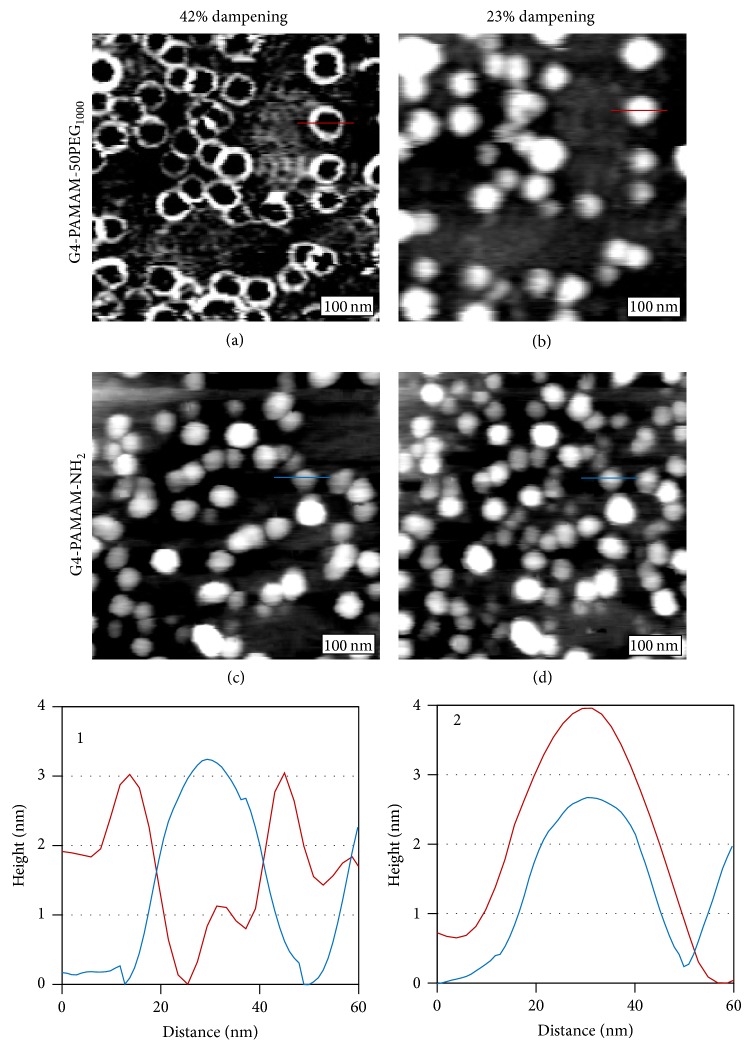
AFM tapping mode imaging of G4-PAMAM-50PEG_1000_ dendrimers. 300 × 300 nm^2^ topographic images of G4-PAMAM-50PEG_1000_ acquired at a damping set point of 42% (a) and 23% (b). 300 × 300 nm^2^ AFM topographic images of G4-PAMAM-NH_2_ at damping set points of 42% (c), and 23% (d). Cursor profile 1 is a representative G4-PAMAM-50PEG_1000_ and G4-PAMAM-NH_2_ dendrimer imaged with 42% dampening as indicated in (a) (red) and (c) (blue). Cursor profile 2 is a representative G4-PAMAM-50PEG_1000_ and G4-PAMAM-NH_2_ dendrimer imaged with 23% dampening as indicated in (b) (red) and (d) (blue).

The characteristic AFM tapping mode images of G4-PAMAM-50PEG_1000_, are shown in Figures 1(a) and 1(b). The two images are of the same area but under two different damping set points during tapping mode AFM imaging. In the case of G4-PAMAM-50PEG_1000_, the topographic images were found to vary with the set point values. In Figure 1(a), at 42% damping, each dendrimer appears as a ring. Reducing the damping to 23%, that is, gentler tapping, an ellipsoidal cap morphology is observed, as shown in Figure 1(b). We have varied damping (from 0% to 100%) and found that dendrimers appear as either rings as shown in Figure 1(a) or ellipsoidal caps as shown in Figure 1(b). We assigned the two features to PAMAM core and PEG shell (or coating), respectively. The assignment is based on three observations. First, the damping-dependence was not present for non-PEGylated dendrimers, such as G4-PAMAM-NH_2_, as revealed in Figures 1(c) and 1(d). Regardless of tapping conditions, the G4-PAMAM-NH_2_ dendrimers always appear as ellipsoidal caps, consistent with prior studies [16]. Secondly, the core region of the G4-PAMAM-50PEG_1000_ ring structures is commensurate in size with that of the G4-PAMAM-NH_2_ core, as shown in cursor 1 of Figure 1. Thirdly, damping dependence in tapping mode AFM imaging typically indicates variation in materials' viscoelastic property, for example, how materials respond to periodical tapping of the AFM tip [24–27]. The distinct differences in the case of G4-PAMAM-50PEG_1000_ dendrimers are consistent with a tight and elastic dendrimer core and a relatively loose and viscoelastic PEG coating. The PEG regions could exhibit high responses at specific damping conditions, revealing rings more evidently.

The width of the rings, measured as full width at half of the maximum height, varies from 5.0 to 12.4 nm, with an average value of 6.9 ± 2.3 nm. The measured ring width provides an approximate view regarding the extension, or conformation, of the PEG molecules suggesting a variation in the PEG conformation. According to AFM imaging, the thickness of the PEG coating surrounding individual dendrimers is asymmetric. The difference between the thickest and thinnest regions of the PEG coating on a single dendrimer varies by 3.4% up to 47.6%. This preliminary assessment of dendrimer morphology indicates that the PEG density and extension are not uniform at the outer shell of individual dendrimers. To determine the fine structure of the PEG coating, STM imaging is performed and provides a more accurate visualization of the PEG presentation, as will be discussed in detail in Section 3.2.

**Figure 2:**
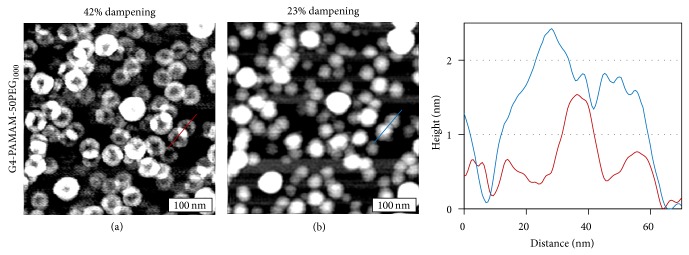
300 × 300 nm^2^ AFM topographic images of G4-PAMAM-50PEG_1000_ imaged with 42% (a) and 23% (b) dampening. Cursor profile is taken from representative G4-PAMAM-50PEG_1000_ dendrimers in images (a) (red) and (b) (blue).

To verify that the features are not due to deformation upon surface immobilization, we have also imaged dendrimers at various surface coverages. At high surface coverage, as shown in Figure 2, AFM topographs are again found to depend on tapping conditions. Overall, the trend is similar to that at low coverage, that is, solid ellipsoidal caps are observed under most tapping conditions, while rings are observed at greater dampening. This indicates that the ring-like features are intrinsic to the dendrimer structure instead of surface deformation.

One specific difference was seen under 42% damping, at which ring contrast was observed previously; the morphology appears fragmented, analogous to flower pedals, as shown in Figure 2(a). The brighter lobes correspond to overlapping PEG regions between two neighboring dendrimers. This overlapping region can extend the entire thickness of the PEG coating up to the PAMAM core as shown in the cursor profile (red). At 23% damping, PEG-coated G4 dendrimers appear as individual ellipsoidal caps with a clear depression between adjacent dendrimers, as shown in Figure 2(b) and the cursor (blue) of Figure 2. In the case of G4-PAMAM-NH_2_ dendrimers, no overlap among dendrimers was ever observed, regardless of imaging conditions. These observations suggest that the PEG chains could be interdigitated at close proximity, which could lead to stacking, interspersion, and merging among neighboring dendrimers. This behavior is in sharp contrast to the PAMAM cores, where no overlapping or coalescing was seen. These observations are important in the context of drug delivery in vivo, as aggregation due to high accumulations of dendrimers is of concern.

**Figure 3:**
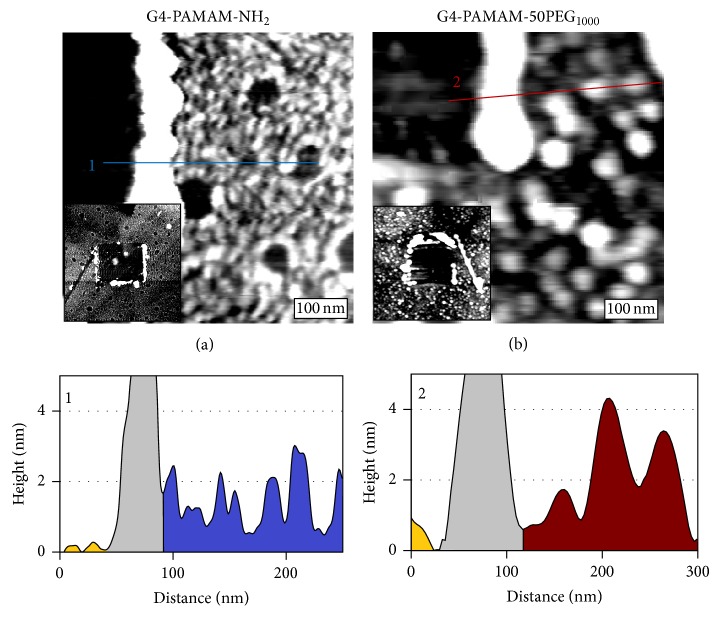
Height characterization of dendrimers without and with PEGylation via nanoshaving. (a) A 300 × 300 nm^2^ AFM topographic image of a nanoshaved monolayer of G4-PAMAM-NH_2_. The (a) inset is a 1.5 × 1.5 *μ*m AFM topographic image of the G4-PAMAM-NH_2_ surface where a 0.5 × 0.5 *μ*m area was removed during nanoshaving to reveal the Au substrate. (b) A 400 × 400 nm^2^ AFM topographic image of a nanoshaved monolayer of G4-PAMAM-50PEG_1000_. The (b) inset is a 1.5 × 1.5 *μ*m AFM topographic image of the G4-PAMAM-50PEG_1000_ surface where a 0.5 × 0.5 *μ*m area was removed during nanoshaving to reveal the Au substrate. Cursor 1 reveals the height of individual G4-PAMAM-NH_2_ from image (a). From left to right, the gold substrate (gold), dendrimers displaced during nanoshaving (grey), and the dendrimer monolayer (blue). Cursor 2 reveals the height of individual G4-PAMAM-50PEG_1000_ from image (b). From left to right, the gold substrate (gold), dendrimers displaced during nanoshaving (grey), and the dendrimer monolayer (red).

Our previous work has demonstrated that the physical height of surface immobilized dendrimers can be measured using nanoshaving, an AFM based technique, in conjunction with topographic imaging [15–17]. The surface was first surveyed to select a relatively flat 1.5 *μ*m × 1.5 *μ*m region. Higher force was then applied during scanning of a central 0.5 *μ*m × 0.5 *μ*m region to remove the dendrimer monolayer. Finally the entire area was imaged again in tapping mode to reveal both the freshly exposed surface region and the surrounding dendrimer monolayer in a single frame, shown in Figure 3(a) inset. The physical height of individual G4-PAMAM-NH_2_ dendrimers relative to the bare substrate can be directly obtained from the cursor profile. As shown in Figure 3(a), cursor 1 reveals the height of five individual dendrimers. The height taken from multiple experiments and a large number of G4-PAMAM-NH_2_ dendrimers was found to be 2.2 ± 0.3 nm, which is identical to 3 previous measurements of surface immobilized G4-PAMAM dendrimers [15–17]. This value is lower than 4.4 nm determined by DLS which probes hydrodynamic diameter. This is not uncommon due to the deformation of the dendrimer upon surface immobilization under ambient conditions [15–17, 28–30]. G4-PAMAM-50PEG_1000_ dendrimers appear taller than the G4-PAMAM-NH_2_ core. Figure 3(b) is a topographical AFM image containing G4-PAMAM-50PEG_1000_. The dendrimers were displaced in the upper left region, enabling accurate height measurements, as shown for two representative G4-PAMAM-50PEG_1000_ in cursor 2. The average height of G4-PAMAM-50PEG_1000_ measured from two experiments and multiple images is 3.4 ± 1.2 nm, which is 1.2 nm taller than the core, G4-PAMAM-NH_2_. As indicated by the increased standard deviation relative to G4-PAMAM-NH_2_, the PEGylated dendrimers have a far greater distribution of height, indicating a greater range of size and/or conformation upon surface immobilization.

### 3.2. High-Resolution STM Imaging of PEGylated G4 Dendrimers

STM provides submolecular resolution characterization of PEGylated dendrimers, revealing the morphology and structure of the PEG chains. With the overall morphology of dendrimers established by AFM, we could use STM to provide a more detailed look at the intramolecular structure especially at PEG region. Although dendrimers are not sufficiently conductive for direct STM imaging, our prior work has indicated that STM conductivity may be enhanced by coordinating metal ions into the dendrimer [15–17]. This work demonstrates that the same approach is effective to enable STM imaging of PEGylated dendrimers.

**Figure 4:**
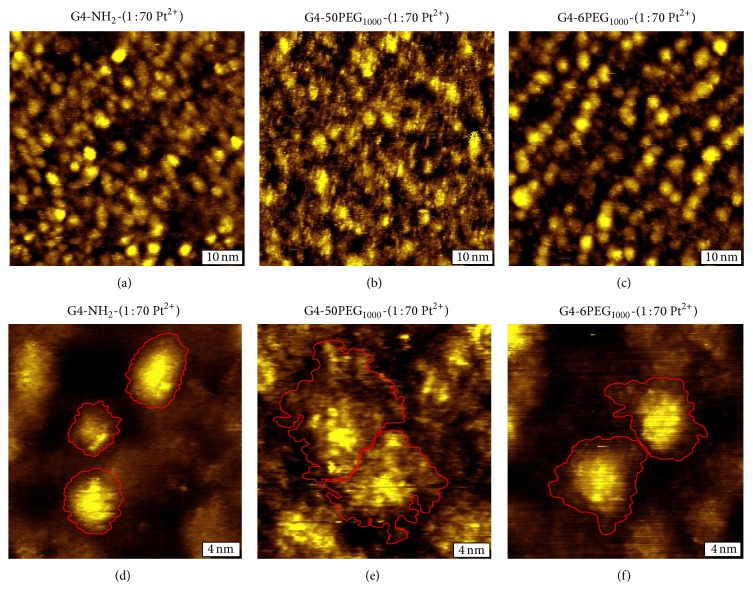
High-resolution characterization of dendrimers with varying degrees of PEGylation. (a) A 60 × 60 nm^2^ STM topographic image of G4-PAMAM-NH_2_. (b) A 15 × 15 nm^2^ STM topographic image of G4-PAMAM-NH_2_ with three representative dendrimer contact areas highlighted in red. (c) 60 × 60 nm^2^ image of G4-PAMAM-50PEG_1000_. (d) 15 × 15 nm^2^ image of G4-PAMAM-50PEG_1000_ with two representative dendrimer/PEG contact areas highlighted in red. (e) 60 × 60 nm^2^ image of G4-PAMAM-6PEG_1000_. (f) 15 × 15 nm^2^ image of G4-PAMAM-6PEG_1000_ with two representative dendrimer/PEG contact areas highlighted in red. All images were acquired at 0.7–0.9 V and 20–30 pA.

The G4-PAMAM-NH_2_ structure was first characterized without PEGylation.  Figure 4(a) is a STM topographic image of G4-PAMAM-NH_2_. Individual dendrimers are clearly resolved, at the coverage of submonolayer with sufficient interparticle separation. At higher resolution shown in Figure 4(b), it is apparent that the geometry of surface immobilized G4-PAMAM-NH_2_ adopts a very similar shape to the most commonly known PAMAM dendrimers, G4-PAMAM-OH, which are ellipsoidal caps [15–17]. While these dendrimers are considered to be nearly spherical in solution, the flattening and deformation from spherical geometry are due to dendrimer-surface interaction. The dendrimer-surface contact areas, as highlighted in red, of the three dendrimers were measured as 7.7, 12.0, and 13.4 nm^2^. Intraparticular features are also visible, such as the bright protrusions on the surface of each dendrimer, which likely correspond to individual NH_2_ termini, analogous to OH termini visualized previously [16].

Upon PEGylation, the STM images reveal significant structural changes from that of core particles.  Figure 4(c) is a typical high-resolution STM image for G4-PAMAM-50PEG_1000_. The PAMAM cores of individual dendrimers are still visible, but the footprint or dendrimer-surface contact area is interspersed with PEG chains spreading out and filling the space between dendrimers. At higher resolution the PEG are visualized more clearly, as shown in Figure 4(d). Unlike G4-PAMAM-NH_2_, the dendrimers are no longer ellipsoidal, as the contact area adopts a more irregular geometry (see red lines tracing the boundaries). The periphery of these dendrimers was determined using multiple cursor profiles to accurately determine the boundaries of each dendrimer. The contact area of the two PEGylated dendrimers is much greater than the corresponding core particles, measuring 41.8 and 29.1 nm^2^, respectively. These high-resolution images reveal that the PEG presentation varies from dendrimer to dendrimer, which leads to a large variation in contact upon surface immobilization. As shown in Figure 4(d), the majority of the PEG chains extend as groups to form PEG “podia.” The PEG podia tend to fan out in regions of bare substrate and bunch up near adjacent dendrimer PEG extensions. These PEG podia extend from 0.5 to 4.5 nm from the core contact region, which indicate variations in PEG conformation. Previous work indicated several conformations adopted by PEG chains attached to solid substrates [9, 10]. To our knowledge, results shown in Figure 4 represent the first observation of the PEG conformational variations in the context of dendrimers.

In order to determine the effect of the packing density of the PEG at the dendrimer surfaces, various PEG : core ratios were prepared.  Figure 4(e) shows a STM topograph of a low PEGylation, G4-PAMAM-6PEG_1000_. The overall morphology of G4-PAMAM-6PEG_1000_ closely resembles that of G4-PAMAM-NH_2_, as clearly defined ellipsoidal caps with intraparticular features and protrusions visible. The degree of spreading observed is shown in Figure 4(f), which falls in between G4-PAMAM-50PEG_1000_ and G4-PAMAM-NH_2_. The dendrimer-surface contact area of the two representative G4-PAMAM-6PEG_1000_ shown in Figure 4(f) measures 24.5 and 21.1 nm^2^, from left to right. The majority of the PEG chains are located close to the core. PEG podia appear shorter than that in high PEG : core ratio cases with their extension 0.5–2.0 nm from the PAMAM core. It is known that the packing density of PEG chains on flat surfaces greatly impacts their conformation [9, 13]. Our investigations indicate that this concept appears to be valid in the context of PEG chains at the surface of PAMAM dendrimers.

The observed coverage dependence of PEG conformation may be understood by taking into account PEG-PEG, PEG-solution, and PEG-surface interactions. On solid flat surfaces PEG_1000_ chains adopt various conformations depending on the interplay between these interactions. Three characteristic conformations have been described previously, known as pancake, mushroom, and brush, with the extension length of 0.5, 1.0, and 2.5 nm, respectively [8–10]. PEG chains on the dendrimer exterior differ from those on flat surfaces due to the curvature and mechanical flexibility of the core. The curvature allows more space than that on flat surfaces under the same coverage, while the relatively soft core allows for greater deformation and flexibility upon surface immobilization. Computational studies on G3-PAMAM-8PEG_1000_ indicate that PEG chains fold in close contact with the dendrimer core in solution, due primarily to interactions between the ether groups of PEG and the protonated amine termini of the dendrimers extending approximately 1 nm in solution [11, 31]. In the case of dendrimers with lower coverage PEG, such as in G4-PAMAM-6PEG_1000_, the PEG chains seem to follow this theoretical prediction and exhibit tightly bound podia. The extension is larger than predicted, extending from 0.5 to 2.5 nm, which indicates the presence of all three conformations described previously. At higher coverage, G4-PAMAM-50PEG_1000_, the PEG podia extension ranges from 0.5 to 4.5 nm. The longest extensions exceed the brush conformation, which we attribute to the effect of dendrimer support and interchain interactions. Considering that PEG-surface interactions could offset the intramolecular interactions in solution phase, local structural characterization is critical to reveal individual dendrimers' structures. The observation of heterogeneity should also be considered when designing dendrimers for drug delivery.

### 3.3. Impact of PEGylation on STM Imaging

**Figure 5:**
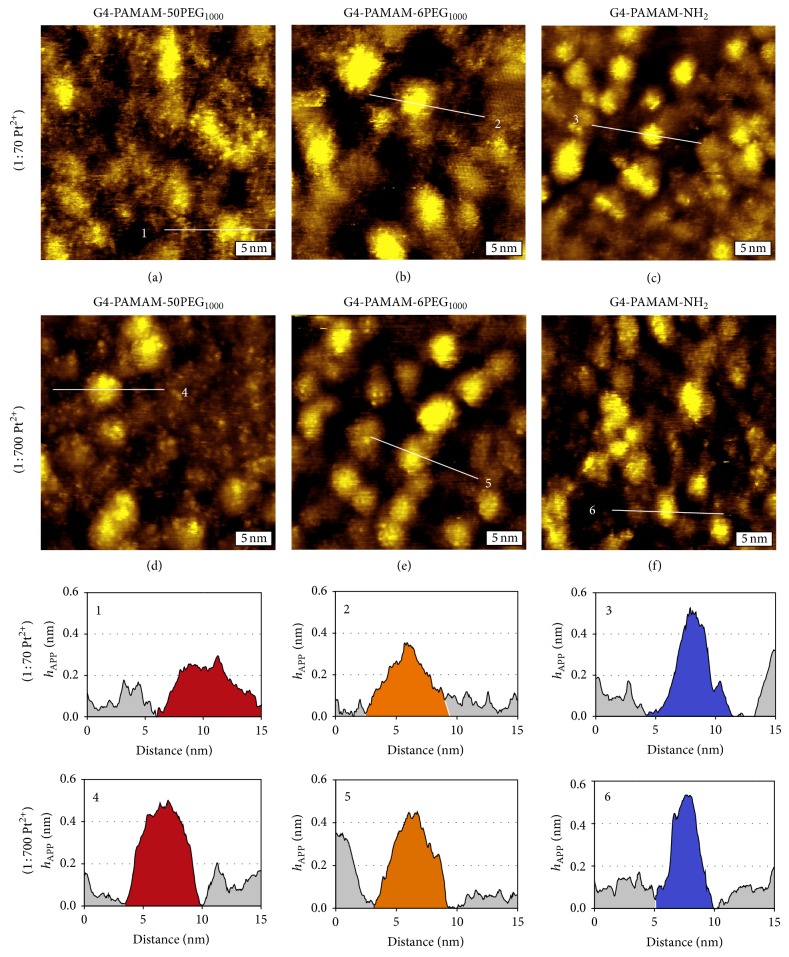
High-resolution characterization of dendrimers doped at 1 : 70 and 1 : 700 dendrimer to Pt^2+^ molar ratios. (a–c) 15 × 15 nm^2^ STM topographic images of G4-PAMAM-50PEG_1000_, G4-PAMAM-6PEG_1000_, and G4-PAMAM-NH_2_ doped in a solution containing a 1 : 70 molar ratio of dendrimer to Pt^2+^. (d–f) 15 × 15 nm^2^ STM topographic images of G4-PAMAM-50PEG_1000_, G4-PAMAM-6PEG_1000_, and G4-PAMAM-NH_2_ doped in a solution containing a 1 : 700 molar ratio of dendrimer to Pt^2^. Cursors 1–3 provide the lateral and apparent height dimensions of individual representative G4-PAMAM-50PEG_1000_, G4-PAMAM-6PEG_1000_, and G4-PAMAM-NH_2_ dendrimers doped at a 1 : 70 Pt^2+^ ratio. Cursors 4–6 provide the lateral and apparent height dimensions of individual representative G4-PAMAM-50PEG_1000_, G4-PAMAM-6PEG_1000_, and G4-PAMAM-NH_2_ dendrimers doped at a 1 : 700 Pt^2+^ ratio. All images were acquired at 0.7–0.9 V and 20–30 pA.

Results from STM investigations indicate that the incorporation of Pt^2+^ ions leads to sufficient conductivity for high-resolution STM imaging, despite PEGylation, which in principle should hinder metal ion doping. In comparison to PAMAM dendrimers, the incorporation of metal ions follows slower kinetics. In the case of G4-PAMAM-OH, for example, each dendrimer was saturated by Pt^2+^ within 48 hrs, under 1 : 70 dendrimer : Pt^2+^ molar ratio, at a 1 *μ*M concentration [16]. The resulting increase in tunneling probability allowed for high-resolution STM imaging, which could resolve individual dendrimer termini [15–17]. Under identical conditions, G4-PAMAM-NH_2_ also yielded high-resolution STM images, as shown in Figures 4(b) and 5(c). In the presence of a PEG coating high-resolution is still provided; however, the apparent height, or *h*_APP_, is lower, as shown in Figures 5(a)–5(c) and cursors 1–3. The average *h*_APP_ of G4-PAMAM-NH_2_ is 0.42 ± 0.11 nm, whereas the *h*_APP_ for G4-PAMAM-6PEG_1000_ and G4-PAMAM-50PEG_1000_ is 0.38 ± 0.13 nm and 0.32 ± 0.07 nm, respectively. We infer that the metal ion concentration inside G4-PAMAM-50PEG_1000_ and G4-PAMAM-6PEG_1000_ dendrimers is lower than G4-PAMAM-NH_2_, leading to a lower local density of states and therefore *h*_APP_. To increase the metal ion coordination within the dendrimers, the dendrimer : Pt^2+^ molar ratio was increased from 1 : 70 to 1 : 700, and the results are shown in Figures 5(d)–5(f) and cursors 4–6. At a higher Pt^2+^ ratio, the average *h*_APP_ of G4-PAMAM-NH_2_, G4-PAMAM-6PEG_1000_, and G4-PAMAM-50PEG_1000_ increased to 0.51 ± 0.07 nm, 0.45 ± 0.12 nm, and 0.56 ± 0.04 nm, respectively. In the case of G4-PAMAM-NH_2_, the average *h*_APP_ increases only 17%, indicating that the intraparticulate Pt^2+^ concentration is very similar under the two concentrations. This is in contrast to G4-PAMAM-50PEG_1000_, where the *h*_APP_ increases by 43%. It is important to note here that a height increase was not observed, as measured by AFM, for any of the dendrimers when the Pt^2+^ ratio was increased. Thus, by increasing the Pt^2+^ concentration, the metal ions were able to penetrate the PEG coating and coordinate with the amines within the dendrimer core to a greater extent. Since the G4-PAMAM-NH_2_ dendrimer is already nearly saturated at the lower Pt^2+^ concentration the effect on *h*_APP_ is relatively small as compared to the highly PEGylated dendrimer. These observations should shed light when using dendrimers as drug delivery vehicles, as the coating may change the pharmacokinetic behavior.

## 4. Conclusions

Using AFM and STM, we have characterized the morphology and structure of PEGylated dendrimers. AFM investigation allows for the visualization of individual dendrimers on surfaces and provides accurate height measurements. In addition, AFM studies reveal that the PAMAM core and PEG shell can be visualized under tapping mode imaging to ascertain the uniformity and distribution of PEGylation on individual dendrimers. Further, the results indicate that PEG chains among adjacent dendrimers could interdigitate, in contrast to the dendrimer cores. STM imaging enables direct visualization of the PEG extensions with high-resolution. The PEG chains at the exterior PAMAM cores adopt various conformations including pancake, mushroom, and brush, similar to that at the solid and flat surfaces. Unique to high coverage PEGylated dendrimers, a greater variation in PEG structure and degree of extension is observed with the PEG podia, up to 4.5 nm from the core. To the best of our knowledge, this work is among the first to reveal high-resolution information on the local structure of PEGylated dendrimers. Collectively, this investigation provides important insight into the structure of coated dendrimers, which shall be important to guide the design and development of better drug delivery vehicles. Work is in progress to correlate structural information with the efficacy of drug delivery.
